# Whole-genome re-sequencing, diversity analysis, and stress-resistance analysis of 77 grape rootstock genotypes

**DOI:** 10.3389/fpls.2023.1102695

**Published:** 2023-02-09

**Authors:** Peipei Wang, Fanggui Zhao, Ting Zheng, Zhongjie Liu, Xinglong Ji, Zhichang Zhang, Tariq Pervaiz, Lingfei Shangguan, Jinggui Fang

**Affiliations:** ^1^ College of Horticulture, Qingdao Agricultural University, Qingdao, China; ^2^ College of Horticulture, Nanjing Agricultural University, Nanjing, China; ^3^ Shandong Zhichang Agricultural Science and Technology Development Co. LTD, Rizhao, China; ^4^ Department of Botany and Plant Sciences, University of California Riverside, Riverside, CA, United States

**Keywords:** grapevine, rootstocks, resequencing, genetic diversity, stress-resistance

## Abstract

**Introduction:**

Grape rootstocks play critical role in the development of the grape industry over the globe for their higher adaptability to various environments, and the evaluation of their genetic diversity among grape genotypes is necessary to the conservation and utility of genotypes.

**Methods:**

To analyze the genetic diversity of grape rootstocks for a better understanding multiple resistance traits, whole-genome re-sequencing of 77 common grape rootstock germplasms was conducted in the present study.

**Results:**

About 645 billion genome sequencing data were generated from the 77 grape rootstocks at an average depth of ~15.5×, based on which the phylogenic clusters were generated and the domestication of grapevine rootstocks was explored. The results indicated that the 77 rootstocks originated from five ancestral components. Through phylogenetic, principal components, and identity-by-descent (IBD) analyses, these 77 grape rootstocks were assembled into ten groups. It is noticed that the wild resources of *V. amurensis* and *V. davidii*, originating from China and being generally considered to have stronger resistance against biotic and abiotic stresses, were sub-divided from the other populations. Further analysis indicated that a high level of linkage disequilibrium was found among the 77 rootstock genotypes, and a total of 2,805,889 single nucleotide polymorphisms (SNPs) were excavated, GWAS analysis among the grape rootstocks located 631, 13, 9, 2, 810, and 44 SNP loci that were responsible to resistances to phylloxera, root-knot nematodes, salt, drought, cold and waterlogging traits.

**Discussion:**

This study generated a significant amount of genomic data from grape rootstocks, thus providing a theoretical basis for further research on the resistance mechanism of grape rootstocks and the breeding of resistant varieties. These findings also reveal that China originated *V. amurensis* and *V. davidii* could broaden the genetic background of grapevine rootstocks and be important germplasm used in breeding high stress-resistant grapevine rootstocks.

## 1 Introduction

Grape rootstocks play critical role in the development of the grape industry over the globe for their higher adaptability to various environments. The first use of resistant rootstocks in the 1870s rescued the grape industry in Europe from the phylloxera disaster, which is a destructive insect pest of grapevines (Viala and Ravaz, 1901; Wang et al., 2019) . Europe and America then continued to research grape rootstocks to improve the resistance of grapes to a variety of biotic and abiotic stresses (Ferris et al., 2012). The use of rootstocks has proven to considerably enhance the resistance of planted types to drought, cold, flood, disease, and insects, as well as broaden the scope of grape cultivation, improve the quality of the grapes, and minimize the pollution produced by pesticides and chemical reagents (Lowe and Walker, 2006; Reisch et al., 2012). With grape phylloxera occurring in many places in the world, and the threat of salt, extreme temperatures, drought, and other ecological environment stresses caused by the deterioration of the natural environment, grape grafting for scion-rootstock seedling propagation has become popular (Riaz et al., 2019). The study of the genetic diversity of rootstocks routinely used in grape production can help us better understand their stress tolerance and expand the genetic resources available for breeding novel rootstocks. Totally, 1,343 rootstocks from 22 countries are registered in the Vitis International Variety Catalogue (VIVC, http://www.vivc.de /), 90% of which are bred through hybridization and show some significant resistance in specific areas, while less than 10% of the rootstocks are seedling offspring of wild varieties. At present, the main varieties globally used in the rootstock breeding programs belongs to *V. rupestris* Scheel, *V. riparia* Michaux, *V. berlandieri* Planchon (accuracy is *Vitis cinerea* (Engelm.) Millardet var. *helleri* (L. H. Bailey) M. O. Moore. According to the writing habit, we continue to use *V. berlandieri* in the manuscript), *V. vinifera* Linn., *V. champini* Planchon, *V. solonis* Llanchon, *V. rotundifolia* Michauzs, *V. amurensis* Rupr., and *V. labrusca* Linn. Among them, those elite genotypes from *V. riparia*×*V. rupestris*, *V. berlandieri*×*V. riparia*, and *V. berlandieri*×*V. rupestris* are the most widely grown (Riaz et al., 2019). Discovering the ancestry of grape rootstocks, describing their genomic and genetic foundation, and applying that information to expand the genetic base of new rootstocks will help to combat developing issues. Genetic diversity research has important theoretical and practical significance for analyzing rootstock resistance, and the availability of the vast grapevine genome would efficiently contribute to these analyses (Liang et al., 2019).

The whole-genome resequencing is one powerful technique that has been widely used to explore crop population genetic variation, locate important trait loci, compare genome sequences, and study the relationships and evolution (Myles et al., 2011; Zhou et al., 2015; Alaimo et al., 2017). Since the grape genome sequence was completed in 2007 (Jaillon et al., 2007; Velasco et al., 2007), a large number of genes related to growth and development, metabolic processes, and biotic and abiotic stress have been discovered, and whole-genome sequencing has been carried out and applied in the explanation of the genomic characteristics that caused phenotypic differences among grapevine varieties and the genetic diversity (Mercenaro et al., 2017; Tabidze et al., 2017; Zhou et al., 2017). Liang et al. (2019) studied the hexaploidization replication event that occurred at an early evolutionary stage, the origin of cultivated grapevine, as well as the genetic diversity based on whole-genome resequencing of 472 Vitis accessions including 12 rootstock genotypes were mostly derived from interspecific hybrids. The cultivated grape population is more closely related to the oriental wild species *sylvestris* and expanded westward after domestication (Mcgovern and Mondavi, 2003). After arriving in Western Europe, the diversity slightly decreased (Mcgovern and Mondavi, 2003; Myles et al., 2011). Before this study, researchers recently conducted resequencing with a small sample size, and they reported 36 grapevine accessions in total (Mercenaro et al., 2017; Tabidze et al., 2017; Zhou et al., 2017). Among them, Mercenaro et al. (2017) and Tabidze et al. (2017) re-sequenced representative cultivars of Sardinian and Georgian grapes, respectively, to explain the genomic characteristics that caused phenotypic differences between these varieties. Therefore, genome-level analysis has not been conducted for a group of individual characteristics in grapes, especially rootstocks, which play an important role in grape cultivation.

With an increasing number of grapevine rootstock genetic germplasms being gathered and used in the grape business, research into their genetic diversity, QTL mapping, and trait heredity change is becoming increasingly important. Here in this study, in order to explain more thoroughly on the genetic diversities and the genetic controlling networks of some important traits of grapevine rootstocks, the rootstock resources of 77 genotypes were re-sequenced to elucidate the genetic relationships between these rootstocks to provide references for research on the resistance mechanisms of rootstocks and breeding improvement.

## 2 Materials and methods

### 2.1 Sample collection

A total of 77 grape rootstocks were used as experimental martial, collected from the Zhengzhou Fruit Research Institute, CAAS, Zhejiang Academy of Agricultural Sciences, Shandong Agricultural University, Zhangjiagang Shenyuan Grape Technology Co. Ltd., and the Changli Fruit Institute, Hebei Academy of Agricultural and Forestry Sciences (**Tables S1-4**), including 3 samples of Muscadinia and 74 samples belonging to 12 species and their hybrids of Euvitis, from North American species (*V. riparia* Michx., *V. rupestris* Scheele., *V. berlandieri* Planchon., *V. labrusca* Linn., *V. champini* Planchon), and Asian species (*V. vinifera* Linn., *V. amurensis* Rupr., *V. dacidii* Foëx., *V. heyneana* Roem. and Schult., *V. pseudoreticulata* W.T. Wang., *V. adstricta* Hance., *V. heyneana* subsp. Ficifolia). These resources have also been used to a certain extent in the breeding of grape rootstocks.

### 2.2 DNA extraction and whole-genome sequencing

The genomic DNA was extracted with the traditional CTAB method from the grape leaves, and the DNA concentration and integrity were detected by agarose gel electrophoresis. The DNA (5 μg) was separated into ~500 bp fragments, and these fragments were used for library construction with the NEBNex DNA Library Prep Reagent Set for Illumina (BioLabs). The sequences were sequenced on an Illumina HiSeq PE150 platform at Beijing Novogene Bioinformation Technology Co. Ltd. The whole genome of each sample was re-sequenced at an average sequencing depth of ~10×.

### 2.3 Genome-wide polymorphism detection

Burrows-Wheeler Aligner (BWA) software (Version: 0.7.10-r789) was used for comparison with the reference genome, with the command ‘mem -t 4 -k -M’ (Li and Durbin, 2009). The single nucleotide polymorphism (SNP) calling was performed on a population scale with a Bayesian approach as implemented in the package SAMtools (Version: 1.3.1) and GATK (version 3.7) (Li et al., 2009). We retained high-quality SNPs, with the minor allele frequency ≥ 0.05, mapping quality ≥ 20, and missing rates < 0.20 for subsequent analysis after filtration. SNP and Indel annotations were performed using the package ANNOVAR (Version: 2015-12-14) (Wang et al., 2010), which includes filter-based annotation, gene-based annotation, region-based annotations, and other functionalities. According to the annotation, SNPs were categorized in exonic regions, intronic regions, upstream and downstream regions, splicing sites, and intergenic regions. SNPs in coding exons were further grouped into synonymous SNPs, non-synonymous SNPs, stop gain, and stop loss.

### 2.4 Phylogenic analysis

An individual-based neighbor-joining (NJ) tree was constructed to clarify the phylogenetic relationship based on the *p*-distance using TreeBeST (version 1.9.2) software (Vilella et al., 2009) and visualized with MEGA (version 6.0) (Tamura et al., 2013). Principal component analysis (PCA) was conducted using Genome-wide Complex Trait Analysis (GCTA, version: 1.25.3) software (Yang et al., 2011) to evaluate the genetic structure, with the significance level of the eigenvector determined using the Tracey-Widom test. The genetics structure was examined with an expectation-maximization algorithm as implemented using ADMIXTURE (Version: 1.3) (Alexander et al., 2009). We predefined the number of genetic clusters *K* from 2 to 8 with 10,000 iterations for each run to explore the convergence of individuals.

### 2.5 Linkage disequilibrium analysis and pedigree construction

The pattern of linkage disequilibrium (LD) was compared using SNPs. The degree of linkage disequilibrium coefficient (*r^2^*) between pairwise SNPs was calculated to estimate LD decay with PopldDecay software (Version: v3.31) (Zhang et al., 2019). ‘-n -dprime -minMAF 0.05’ was set in the program. The average *r^2^* was calculated in a 500-kb window and averaged in the whole genome, and LD decay figures were drawn with an R script. The IBD was calculated for all of the pairwise comparisons among the 77 rootstocks with PLINK (Purcell et al., 2007). Pairs of accessions were considered to be genetically identical if IBD > 95%, and the cut-off value for first-degree relatives (IBD value ≥ 0.45) was determined according to the distribution pattern of all of the pairwise IBD values. The network images based on IBD were constructed using Cytoscape (Version 3.6.0). Pairwise *F_ST_* values (Weir and Cockerham, 1984) were computed in the same windows to measure the population differentiation between groups.

### 2.6 GWAS

The phenotypic traits of 77 grape rootstock samples were investigated, and their germplasm resources of resistance to phylloxera, root knot nematode, salt tolerance, cold tolerance, drought tolerance, and waterlogging tolerance were measured. Combined with these phenotypic data and based on SNP markers, a mixed linear model (MLM) in GEMMA software was used to analyze the resistance traits, such as phylloxera, root knot nematodes, salt, cold, drought, and waterlogging, in the GWAS analysis. We screened the potential candidate SNPs based on the associated significance (*P*-value).

## 3 Results

### 3.1 SNPs and genomic structural variations

About 645 billion genome sequencing data were generated from the 77 grape rootstocks at an average depth of ~15.5×, from which a total of 4,269,582,820 clean reads and 3,943,015,251 mapped reads were acquired. The mapping rate of these raw reads to the *V. vinifera* reference genome (BioProject: PRJNA673645) was 92.19% (**Tables S1, 1**), and the estimated error rate was 0.03% (**Tables S1, 2**). The quality of the sequencing was high (Q20 ≥ 94.52, Q30 ≥ 88.27), and the GC distribution was normal. A total of 2,805,889 SNP sites were identified in this research (**Tables S1–4**).

### 3.2 Analysis of grape rootstock phylogeny

**Figure 1:**
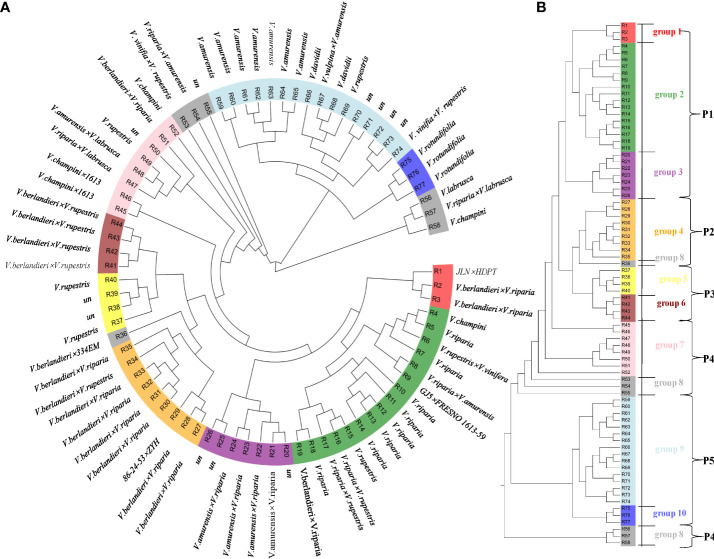
Phylogenetic analysis of 77 rootstocks samples. **(A)** Neighbor-joining (NJ) phylogenetic tree of grapevine accessions inferred from the whole-genome SNPs with 100 nonparametric bootstraps. **(B)** Five populations of 77 rootstock samples belonging to 10 groups.

According to our findings, the 77 rootstock samples were spread over the phylogenetic tree’s (**Figure 1**) ten main branches and belonged to five distinct populations: the Chinese and American group, *V. riparia* group, *V. berlandieri* group, *V. rupestris* group, and highly hybrid group (**Figure S1**; **Table S1-5**). Most of the individuals in Population 1 (P1) are variants of *V. riparia* and their hybrids with other populations, such as R19, R2, R3 (*V. berlandieri*× *V. riparia*), R16, R17 (*V. rupestris*× *V. riparia*), R22, R8 (*V. amurensis*× *V. riparia*), R10, and R11 (*V. riparia*). This group also contains one variety of *V. champini* (R4) and one variety of *V. rupestris* (R15). P1 contains three groups (group1, group2, and group3) that are closely related to each other, indicating that *V. champini*, *V. rupestris*, and *V. riparia* have a close genetic relationship; group4 and R36 from group8 are the offspring of *V. berlandieri*× *V. riparia* and are classified as Population 2 (P2); Population 3 (P3) includes group5 and group6, which represent *V. rupestris* and its hybrid offspring with *V. berlandieri*; group5 belongs to *V. rupestris*; and group6 belongs to *V. berlandieri*× *V. rupestris*, and P4 represents a relatively complex group with relatively distant genetic relationships between *V. labrusca* (R56) and its hybrid offspring with other populations of R57, R45, R46, R47, and R48, *V. champini* (R52 and R58), the offspring of *V. riparia* (R50, R51, R53, and R54), and individuals of unknown parental origin (R55); and P5 has obvious regional characteristics and is divided into *V. rotundifolia* of American origin and the wild resources of China.

**Figure 2:**
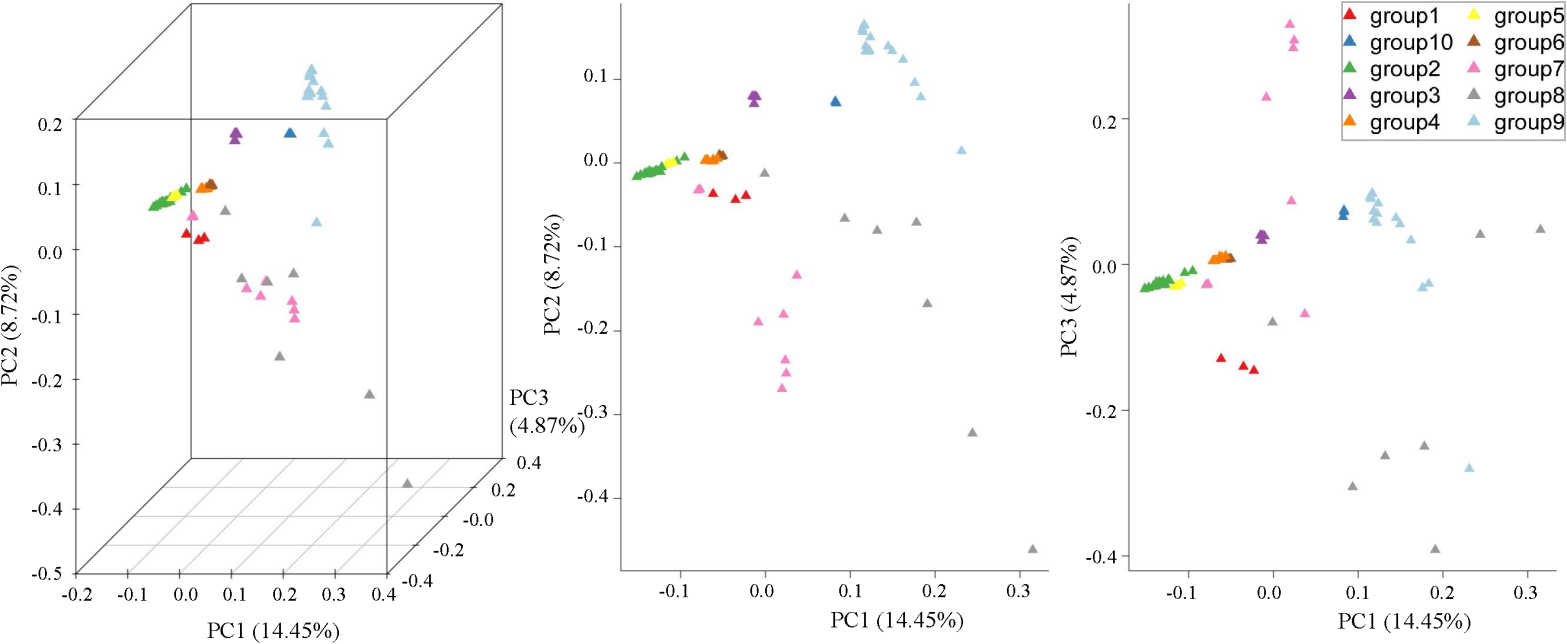
PCA plots of the first three components of 77 grape rootstocks using whole-genome SNP data.

The evaluation of their above genetic similarity and clustering relationships through PCA indicated that the proportions of the total variance of PC1, PC2, and PC3 were 14.45%, 8.72%, and 4.87%, respectively, furthermore a total of 28.04% of all the genetic differences were observed. The first principal component (PC1) separated groups 2, 3, 4, 5, 6, 9, and 10, which had differences at the parental and geographic levels (**Figure 2**). Group 5 was clustered in group 2 and clearly separated from other groups. Group 4 and group 6 clustered together due to the common ancestor i.e. *V. berlandieri*. The group1, group2, and group3 descendants of *V. riparia* were scattered and be separated by PC2. The dispersion between the individuals in group7 and group8 was more apparent, it shows that genetic differentiation existed in each group, with large genetic differences and rich genetic diversity. Group9 and group10 were situated far away from the other populations. The results of PCA were also consistent with the phylogenetic clusters.

**Figure 3:**
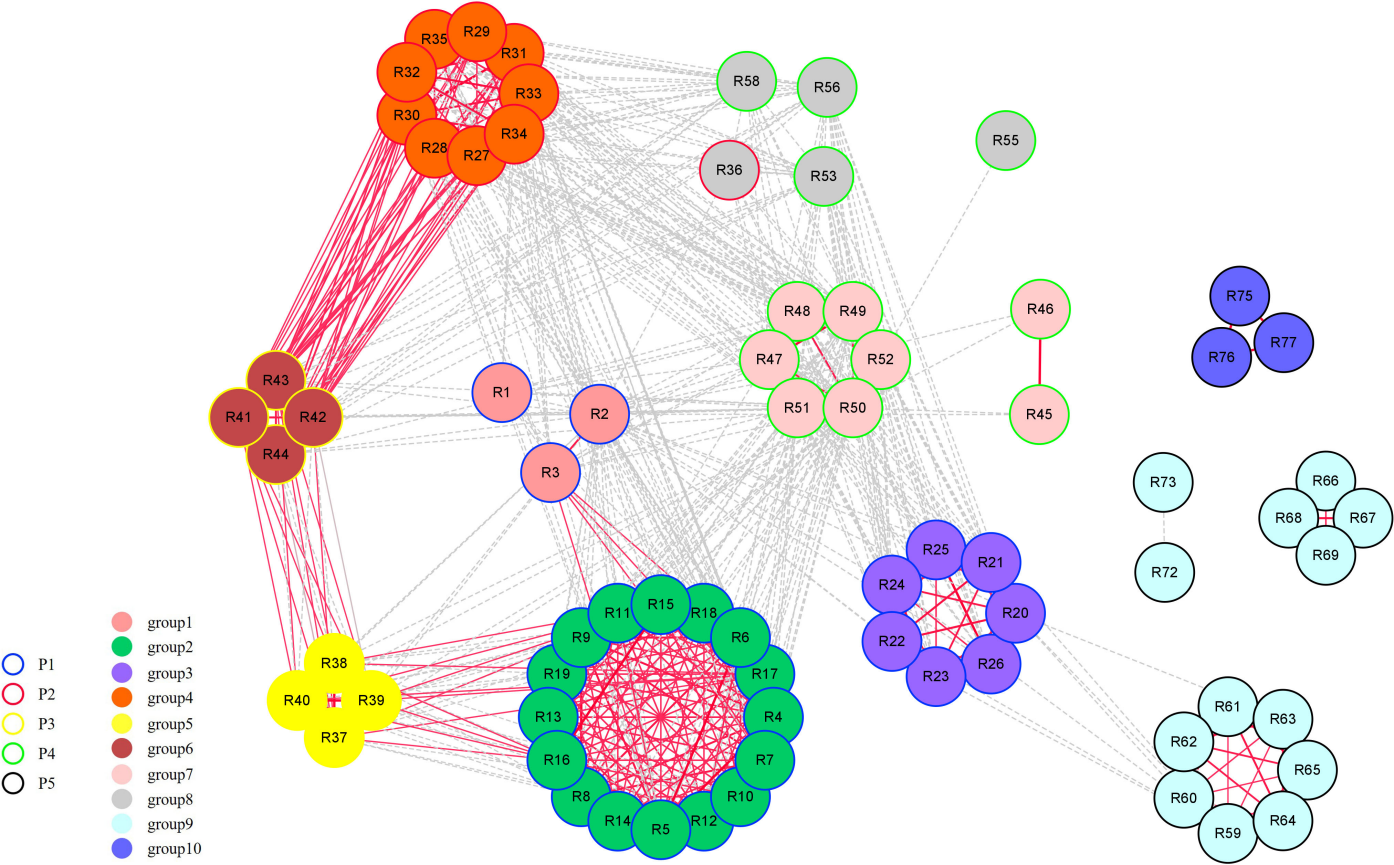
First-degree relationship network of 74 rootstock accessions. A dashed line represents an identity-by-descent (IBD) value between 0.45 and 0.50 (**Figure S2**). A solid line represents an IBD value equal to or greater than 0.50. The thickness of the line is proportional to the calculated IBD value.

Further pedigree analysis within grape rootstock groups based on identity-by-descent was also conducted. the majority of the first-degree relationships were among accessions in the same major *Vitis* categories (**Figure 3**). Group2, group4, group5, and group6 are closely related to each other, forming a compact independent cluster individually, which is the result of the hybridization of *V. rupestris, V. riparia*, and *V. berlandieri*. Group3 and group9 have the common parent *V. amurensis*, and are weakly related, however, P5, with obvious regional characteristics, formed scattered clusters.

### 3.3 Analysis of linkage disequilibrium

**Figure 4:**
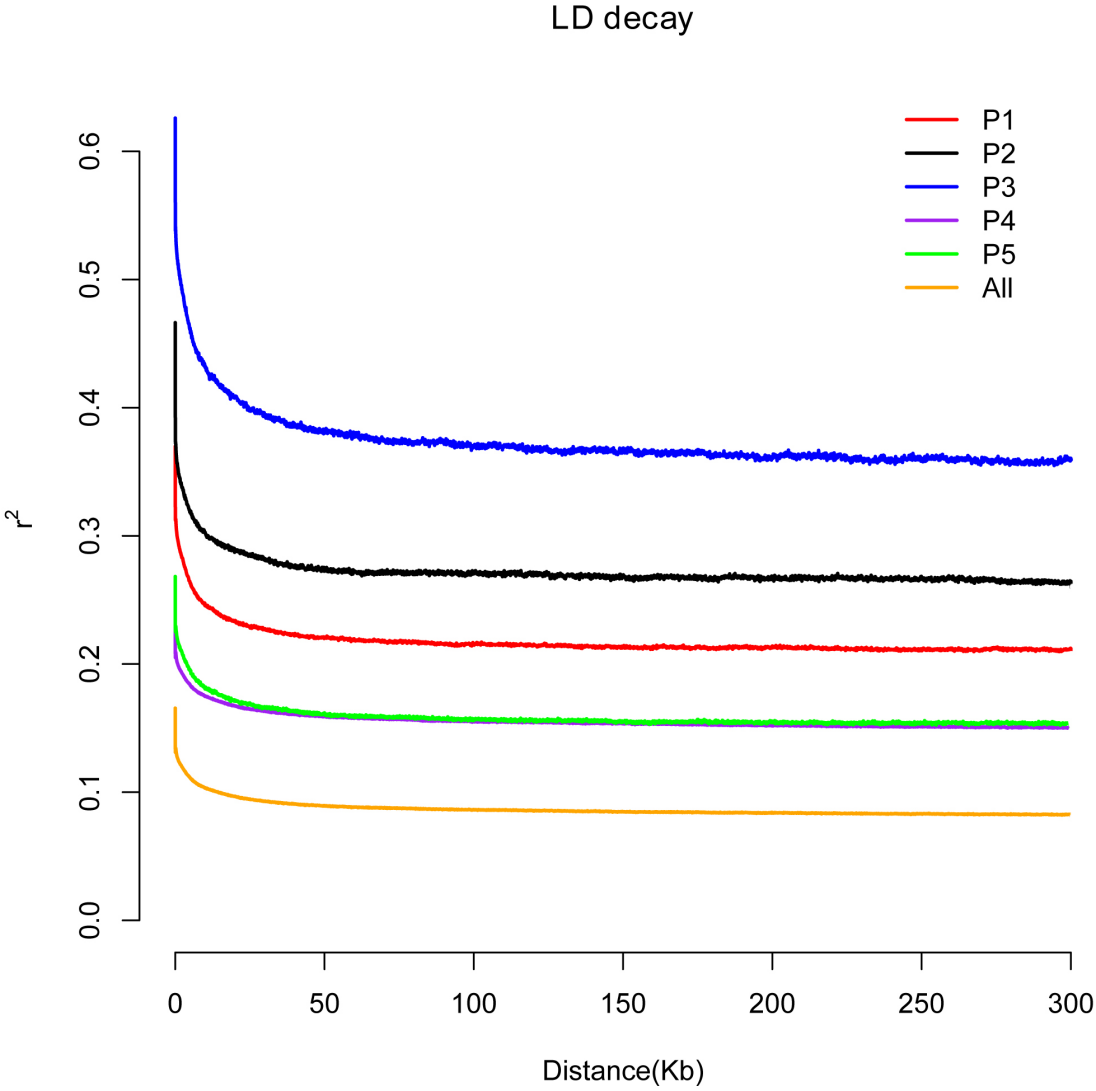
Decay of linkage disequilibrium of the rootstocks of different grape varieties. The abscissa represents the distance of linkage disequilibrium (LD), and the ordinate is the correlation coefficient of linkage disequilibrium.

In plant-forward genetics studies, determining the linkage disequilibrium (LD, represented as r2) pattern is critical. The population size of the species, fertility (selfing or hybridization), selection pressure, and the rate of recombination all affect the level of LD, for which r^2^ between the paired SNP sites were calculated using the available genome-wide SNPs. It was found that the attenuation of LD was 4.6 kb in P1, 6.1 kb in P2, 23.2 kb in P3, and 4 kb in P4, reaching half of the maximum r^2^. These parameters are substantially greater than Liang et al. (2019) and Kui et al. (2020) identification of 2.9 kb of European wild grapes and 350 bp of cultivated grapes. The LD attenuation distance of *V. amurensis* from China and *V. rotundifolia* from the United States was 1.7 kb, which is smaller than that of wild European grapes. The r^2^ was calculated uniformly for all of the rootstock species resources, and the results showed that an increase of 2 kb physical distance could reach half of the maximum r^2^, and an increase of 5.1 kb attenuated to 0.1 (**Figure 4**). The rate of LD decay differed in the different populations. The attenuation speeds of P4 and P5 were the same, whereas the remainder were in the order of P2 > P1 > P3. The LD of P4 and P5 had the lowest attenuation distance, which might be due to the significant genetic variation among these two groups. The *V. rupestris* population P3 had the slowest attenuation rate, the most domestication, and the most intense selection. The average coefficient size of LD at a distance of 50 kb in the genome was.41, but was reduced to 0.37 when reaching 100 kb.

Combined analysis with ADMIXTURE (**Figure S3**, **S4**) was carried out, and when K=2 was selected, group9 could be clearly distinguished from the other populations. Group4 and group6 are both hybrid descendants of *V. berlandieri*, and their composition was the same and could be distinguished when K=4. The composition of group2 and group5 was the same, which suggested that *V. rupestris* and *V. riparia* had a common ancestor, and their differentiation was observed until K=5. When K=3, group10 and group3 could be differentiated; when K=4, group8 was highly polymorphic and had a distant genetic distance from the other populations; when K=5, group7 was differentiated; and when K=6-8, more groups appeared in group7, 8, and 9.

### 3.4 Selection signals in the 77 grape rootstocks

**Figure 5:**
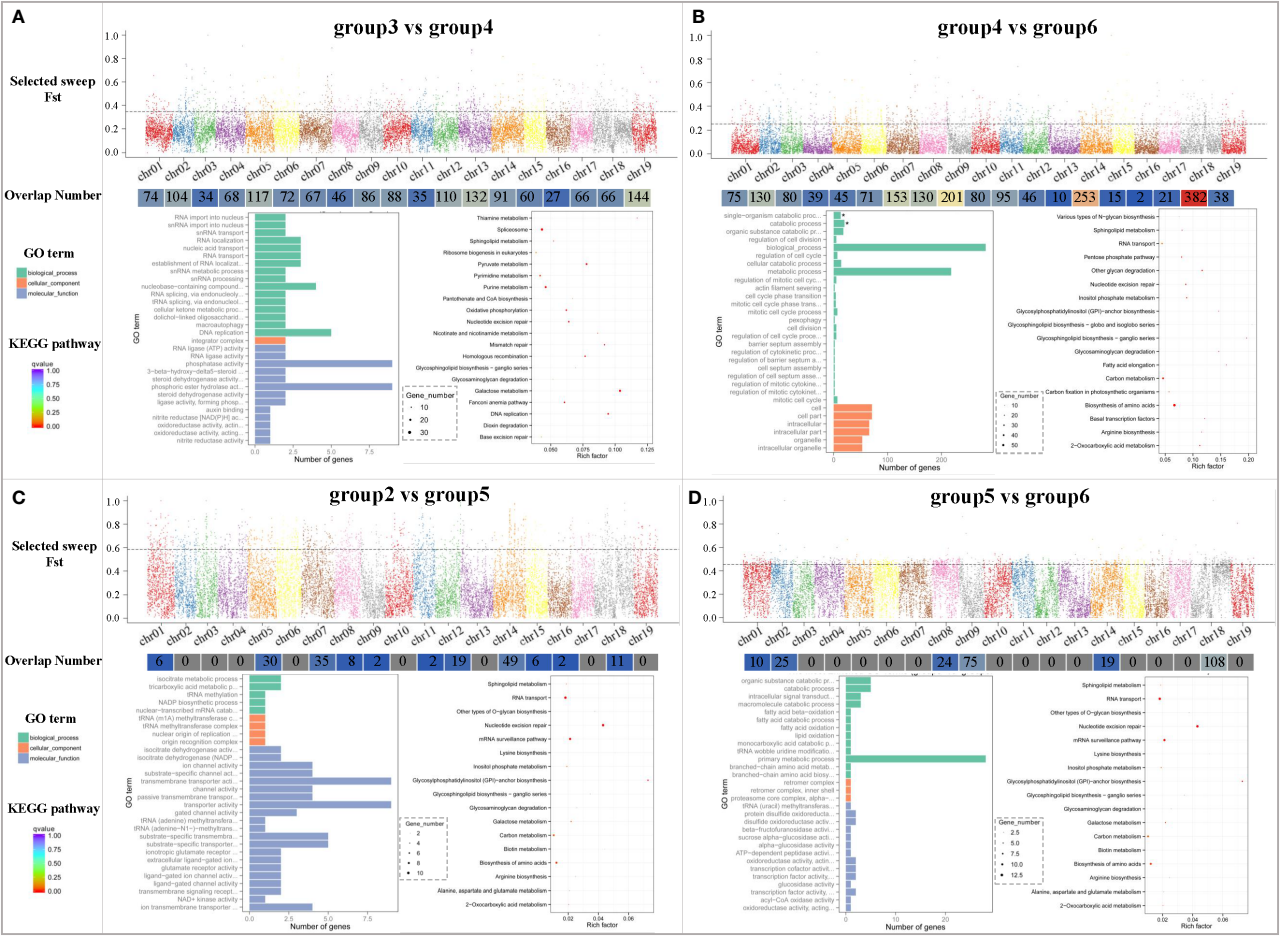
Selective sweep regions and gene enrichment analysis of major groups. **(A)** group3 vs. group4; **(B)** group4 vs. group6; **(C)** group2 vs. group5; and **(D)** group5 vs. group6. Each group includes the Fst distribution map (*X*-axis represents different chromosomes, *Y*-axis represents the Fst value in the corresponding chromosome window, and the dotted line represents the selection threshold top 5%); overlap number (*X*-axis represents different chromosomes, and *Y*-axis represents the number of corresponding overlaps); and GO term and KEGG pathway enrichment of the candidate selective sweep genes. The size of the bubble represents the number of genes in the corresponding GO category. The color of the bubble shows the corresponding *P*-value. Rich factor shows the percentage of enriched genes out of the total number in the GO category.

The fixation index (*F_ST_*) can measure the degree of difference across populations by reflecting the allelic heterozygosity level of a population. The Fast of group4 and group6 was about 0.25, indicating that the genetic differentiation of these two groups was relatively large, whereas the other groups were greater than 0.25, indicating that there was strong genetic differentiation among these populations. As indicated in **Figure 5**, the highest Fst value appeared between group2 and group5, indicating that there was relatively high genetic differentiation between *V. rupestris* and *V. riparia*, whereas the lowest level of genetic differentiation was observed in group4 and group6. Group4 was closer to group6 and relatively far from the group3, which was consistent with the results of the PCA and phylogenetic analysis. Overall, the Fst between the populations was greater than exceeded0.25, indicating that the high level of genetic diversity among the grape rootstocks. Counting of the overlap number indicated that the largest number of gene fragments was enriched in group3 vs. group4, distributed on 19 chromosomes. The Gene Ontology (GO) analysis terms were mainly enriched in biological process and molecular function, including nucleobase-containing compound, DNA replication, phosphatase activity, phosphoric ester hydrolase activity, spliceosome and galactose metabolism, and purine metabolism. The genes of *V. riparia* × *V. berlandieri* and *V. rupestris* × *V. berlandieri* were distributed on 19 chromosomes after selective elimination, especially chr18, and they were mainly involved in biological process and cellular components, and the pathways mainly included biosynthesis of amino acids and carbon metabolism. Group2 vs. group5 represents *V. rupestris* and its hybridization with *V. riparia*, and the hybridization resulted in differences in genes on 11 chromosomes, which mainly affected molecular function, as well as inositol phosphate metabolism, carbon metabolism, and biosynthesis of amino acids pathway. Group5 vs. group6 represents *V. rupestris* and its hybridization with *V. berlandieri*, which caused genetic differences on six chromosomes, mainly affecting the biological process, RNA transport, mRNA surveillance pathway, and nucleotide excision repair pathways.

**Figure 6:**
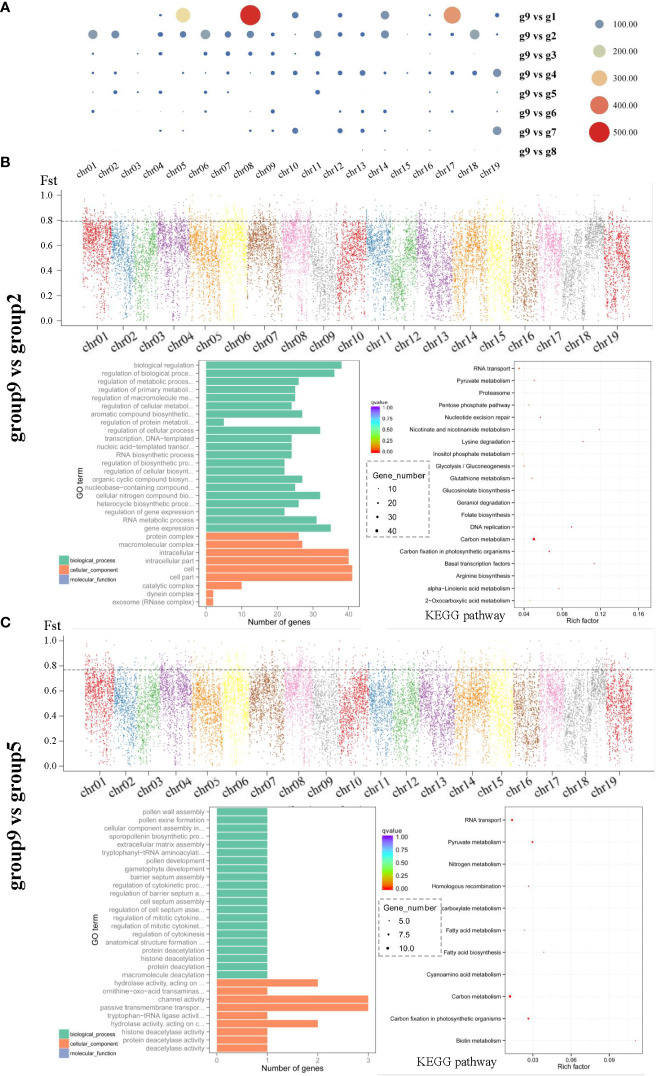
Selection elimination and gene enrichment analysis of the Chinese population and other groups. **(A)** Overlap Number (the x-axis represents different chromosomes, and the y-axis represents the number of corresponding overlaps). **(B, C)** group9 vs group2, group9 vs group5, Fst distribution map of eight groups (the x-axis represents different chromosomes, the y-axis represents the Fst value in the corresponding chromosome window, and the dotted line represents the selection threshold top 5%); and GO term and KEGG pathway enrichment of the candidate selective sweep genes (the size of the bubble represents the number of genes in the corresponding GO category, the color of the bubble shows the corresponding *P-*value, and the rich factor shows the percentage of enriched genes out of the total number in the GO category).

To analyze the degree of differentiation between Chinese and other populations, we performed selection and removal analysis of group 9 and other groups, and the degree of differentiation was in the following order: group 8, group 3, group 7, group 4, group 6, group 1, group 5, and group 2 (**Figure 6**, **S5**). Most individuals in group9 belonged to *V. amurensis*, which had a relatively low degree of differentiation between group 3 and group 8 (which also contain components of *V. amurensis*), while a higher degree of differentiation was detected in the seedlings of *V. riparia* (group 2) and *V. rupestris* (group 5). The biological process category had the most significant difference in GO functions between group 9 and the other groups. Group 9 had more overlaps with group 1 and group 2, among which group 2 was relatively evenly distributed on 19 chromosomes (**Figure 6A**). It is worth mentioning that *V. rotundifolia* from the United States belonged to a different subgenus from the other populations and exhibited no genetic differentiation.

### 3.5 GWAS analysis

**Figure 7:**
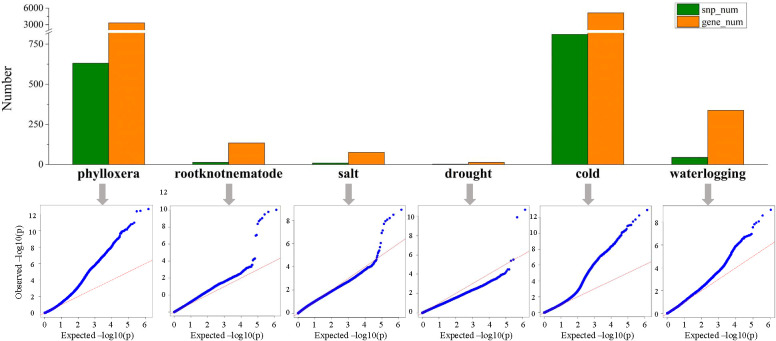
Comparison of the results of the association analysis of six resistance traits. Comparison of the number of associated SNPs and genes; QQ-plot based on the MLM model shows the distribution of actual *P*-values and the expected *P*-values of the non-relevant null hypothesis.

**Figure 8:**
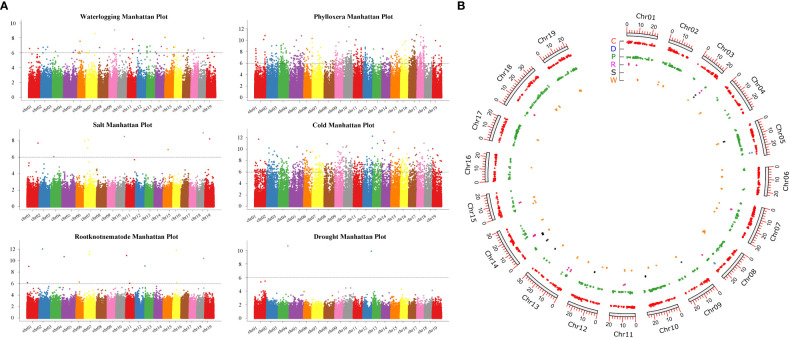
Genome-wide association analysis of six resistance traits. **(A)** The distribution of the *P*-values of the whole genome on the chromosome. The genetic marker effect value is the *P*-value of the whole genome after the F-test is sorted according to the physical location on the chromosome. The *X*-axis represents the genome coordinates, and the *Y*-axis represents log_10_P. The smaller the *P*-value, the stronger the correlation, which is expressed as a larger ordinate. **(B)** The distribution of the located genes on the chromosomes, P: phylloxera, R: root-knot nematode, S: salt, D, drought, C: cold, W: waterlogging.

GWAS analysis were carried out through filtering and screening the genomic data to assess the resistance of grape rootstocks to phylloxera, root-knot nematodes, salt, drought, cold and waterlogging traits, and the locations of 631, 13, 9, 2, 810, and 44 SNP loci, related to 3312, 134, 75, 13, 5133, and 337 genes were identified (**Figure 7**; **Table S2**). Among them, the genes related to resistance to phylloxera, waterlogging and cold were scattered on each chr. Drought-related genes were distributed on chr5 and chr10, with 9 and 4 genes mapped, respectively. Furthermore, the genes related to salt stress were distributed on chr14, chr03, chr10, chr13, chr5, chr9, and chr12. And most genes related to root-knot nematodes were distributed on chr13 and chr14, with 32 and 26 genes mapped respectively (**Figure 8**; **Table S2**). We discovered that the association analysis result for salt was the closest to the predicted P-value of the non-associated null hypothesis using a quantile-quantile (Q-Q) plot, showing that the features were not produced by group stratification. The *P*-value of phylloxera and cold differed the most from the expected *P*-value, indicating that population stratification and individual kinship had a great influence on the association analysis and could easily result in false-positive associations, so the most SNP sites are located.

A total of 13 SNP loci were related to grape rootstock resistance to root-knot nematode in a GWAS analysis, with chr5 accounting for roughly 20% of the total. Furthermore, three were located on serine/threonine-protein kinases containing LRR structure, two PP2 proteins, one cysteine protease *XCP1* gene, one leucine-rich protein kinase, one *MYB48* transcription factor, one *bHLH61* transcription factor, one BTB/POZ domain-containing protein, and one NRT1/PTR FAMILY 5 protein. These genes are associated to plant responses to biotic and abiotic stress, either directly or indirectly. Among the 75 genes associated with salt damage traits, multiple cysteine-rich receptor-like protein kinases are located on chromosome 8, and sodium/hydrogen exchanger 2 is located on chromosome 5. A total of 13 genes were associated with cold tolerance traits, of which nine genes are located on chr2, *P4H1, PPR, PRN, SAD2*, and *Ku70*, and four genes have unknown functions, meanwhile, four genes are located on chr10, *CAT2, PLGG1, PLAT*, and *BTB/POZ-MATH*. A total of 44 SNP loci were mapped to the waterlogging tolerance traits of rootstocks, among them chr3 and chr10 genes hosted the largest number of them. We mapped *WRKY20, WRKY72*, and *WRKY32*, two *MADS-box* genes including *MADS-box 3* and *MADS-box X1*, three F-box genes involved in abiotic stress, one ERF gene and one EIN gene involved in the ethylene regulation, and six endo-1,3 (Reisch et al., 2012)-beta-glucanase genes also involved in plant resistance to disease.

The *P*-values of the GWAS analysis of the traits of grape resistance to phylloxera and cold differed from the ideal *P*-values. We located six WRKY transcription factors related to phylloxera and three genes related to root growth, including ROOT INITIATION DEFECTIVE 3, ROOT PRIMORDIUM DEFECTIVE 1, and SODIUM POTASSIUM ROOT DEFECTIVE 2, which may be related to phylloxera infestation during root development, thus endangering grape growth. C-repeat binding factor 1 was associated with cold-tolerance traits on chr11, which is the master switch of plant cold-resistant crop mechanisms. In addition, we identified 22 MYB transcription factors and found PIF4 and cold shock proteins (CSP2) on chr13. CSP can enhance the ability of cells to resist cold shock stress.

In addition, the association analysis revealed that several attributes shared a large number of the same genes. 19 genes were associated with three traits, of which six genes were also associated with the resistance to phylloxera, salt, and cold traits, including pyruvate kinase isozyme A, armadillo repeat-containing protein LFR, and Very-long-chain 3-ketoacyl-CoA synthase. Six genes were instantaneously and associated with the resistance to phylloxera, cold, and root-knot nematode traits, including three serine/threonine-protein kinases, Protein LURP-one-related 5, and ethanolamine-phosphate cytidylyltransferase. Seven genes relating to resistance to waterlogging, phylloxera, cold, and waterlogging traits were linked, including aluminum-activated malate transporter 12, protein ABCI7, and Ribulose bisphosphate carboxylase/oxygenase activase 1, and two protein phosphatase 2C genes.

## 4 Discussion

Rootstocks plays a pivotal role to protect grapevines from biotic and abiotic stresses including phylloxera, nematodes, viruses, limestone-based soils, salinity, and drought (Riaz et al., 2019). In this study, 77 grape rootstocks currently in cultivated for grape production were re-sequenced. Phylogenetic analysis inferred that the common rootstock varieties were mostly from *V. riparia*, *V. rupestris*, and *V. berlandieri*, which also confirms the results of Riaz, Pap, Uretsky, Laucou, Boursiquot, Kocsis et al. (Riaz et al., 2019) and Heinitz, Uretsky, Peterson, Huerta-Acosta,Walker (Heinitz et al., 2019) that the world’s existing rootstocks have a narrow genetic basis. Through population genetic structure analysis, we found that 77 rootstocks were mainly derived from five ancestral components. The rootstocks that were first discovered were *V. riparia* and *V. rupestris*, which can be grafted to grapes well. Later, the planting advantage of *V. berlandieri* in Europe was discovered (Viala and Ravaz, 1901; Foëx, 1902), following which extensive rootstock breeding took place (Laucou et al., 2008; Garris et al., 2009; Bavaresco et al., 2015; Pavloušek, 2015; Riaz et al., 2019). This is similar to the genetic structure of cultivated grapes and is the result of limited hybridization between superior varieties, forming a large and complex pedigree (Bacilieri et al., 2013). The various subgroups of rootstocks began to grow before 1.5 mya and proceeded to distinguish and hybridize, resulting in a rise in the size of the group, according to this study. Each group has experienced at least one group expansion and a population bottleneck event.

Researchers have been exploring the origin and evolution of various types of grapes (Aradhya et al., 2003; This et al., 2006; Myles et al., 2011; Bacilieri et al., 2013; Marrano et al., 2018; Liang et al., 2019). The cultivated grapevine has broad genetic variation (This et al., 2006; Marrano et al., 2017). The narrow genetic basis in viticulture may have a significant negative impact on its ability to withstand evolving pests and diseases and climate change (Ollat et al., 2015). In this study, it was found that Chinese wild grapes, especially *V. amurensis*, have a genetic background that is far from the traditional rootstock varieties, have an irreplaceable role in resistance to abiotic stress, and are a good parent choice.

Because of wide adaptability and strong resistance to biotic or abiotic stress (Zhang et al., 2012; Liu and Hua, 2013), wild grapes are an important gene pool for broadening the genetic basis and cultivation of rootstock grapes (Wan et al., 2008; Tian et al., 2008; Heinitz et al., 2019). As a precious germplasm resource, wild grapes play an important role in the Chinese grape industry (Wan et al., 2008; Jiang et al., 2015). In this study, we selected the main wild grape resources in China, including *V. amurensis*, *V. quinquangularis*, *V. davidii*, and *V. pseudoreticulata*. The genetic distance between Chinese wild populations and other populations was farther but closer to *V. rotundifolia*. The Chinese wild grapes were highly differentiated from the seedlings of *V. riparia* (group2) and *V. rupestris* (group5). At present, Chinese breeders have used wild resources such as *V. amurensis* and *V. davidii* for hybridization and have selected excellent rootstock resources (Zhang et al., 2009). Group3 in this study was obtained by hybridization between *V. amurensis* and *V. riparia* and inherited the cold-resistance characteristics of *V. amurensis*, which also confirmed the feasibility of Chinese wild resources as rootstock parents. Further research should be aimed at overcoming the obstacles of interspecies hybridization; hybrid offspring with high resistance, distant genetic relationships, and large genetic differences should be obtained; and the genetic basis of rootstocks should be expanded. This is an important measure for ensuring the sustainable development of the grape industry (Riaz et al., 2019).

Suitable rootstocks are selected according to the demands of the environmental conditions in different regions. Locating genes that control rootstock resistance-related traits is the basis of genetic breeding. This can further analyze the genetic mechanism of resistance and provide an important theoretical basis and genetic resources for further research on rootstock resistance improvement and molecular marker-assisted breeding (Migicovsky et al., 2017; Su et al., 2019). The LD of grape rootstocks revealed a fast deterioration trend in this study, showing that association analysis for highly heterozygous fruit tree crops like grapes required very rich SNP marker data that can cover the whole genome (Migicovsky et al., 2016). GWAS analysis revealed that 3312 and 5133 genes were related to phylloxera and cold stresses, while 19 genes were shared by at least three stress traits (**Table S2**). Members of the serine/threonine kinase family are regarded as the central unit linking hormone and environmental stimuli to changes in metabolism and gene expression (Hardie, 1999; Liang et al., 2019). In this study, we associated a large number of members of the serine/threonine kinase family with the phylloxera, cold, and root-knot nematode stress traits (**Table S2**). As one of the largest transcription factor families in plants, WRKY is one of the transcription factor families that is a key participant in resistance against waterlogging damage (Meng et al., 2016; Su et al., 2019), cold damage (Guo et al., 2014), drought (Meng et al., 2016), phylloxera (Wang et al., 2019), and other external stresses (Guo et al., 2014). We also associated a large number of members of the WRKY gene family with resistance to phylloxera and waterlogging tolerance. In addition, three root development-related genes were associated with resistance to phylloxera, and two MADS-box and three F-box genes were associated with waterlogging, which are closely related to plant abiotic stress. BTB/POZ-MATH has been linked when drought traits are analyzed to interact with ERF/AP2 in response to drought stress and salt stress (Weber and Hellmann, 2009). ABA-mediated Importin beta-like SAD2 has also been shown to be involved in the resistance to cold, salt, and other abiotic stresses (Verslues et al., 2006). Due to the influence of group stratification and kinship, the cold-tolerance association analysis obtained many false-positive results. However, we were interested in the transcription factor CBF (CRT/DRE-binding factor), which is the master switch for plants to improve cold tolerance, and the transcription factor MYB15, which is involved in the regulation of cold tolerance (Agarwal et al., 2006). This may be related to the cold stress-related regulatory module of CBFs-PIF3-phyB recently discovered by Jiang, Shi, Peng, Jia, Yan, Dong et al. (Jiang et al., 2020).

Global disease caused by root-knot nematodes seriously threatens grape production (McKenry and Anwar, 2006), and *V. rotundifolia* is highly resistant to root-knot nematodes (Esmenjaud and Bouquet, 2009). The presence of LRR domains in genes has been linked to biotic stress tolerance in studies. After infection, the number of LRR domains is inversely proportional to the amount of expression. Plant resistance to root-knot infection can be altered by WRKY and bHLH, which control genes encoding LRR (Song et al., 2018). In this study, we found four genes containing the LRR structure that was also related to the transcription factors MYB48 and bHLH61, which may be related to the role of the LRR domain. In addition, we found that the resistance of grapes to root-knot nematodes was related to HMGR and PP2. HMGR is activated during root-knot nematode infection and causes a large number of alkaloids and terpenoids to be synthesized, thus improving the defense ability of grapes (Cramer et al., 1993). Furthermore, PP2 is a dimeric chitin-binding lectin that can maintain plant morphology and protect wounds (Zhang et al., 2011; Guo et al., 2018), and its F-box domain can also participate in protein degradation (Guo et al., 2018). Overexpression of PP2 can inhibit the feeding habits of the green peach aphid (Zhang et al., 2011). The cysteine protease XCP1 on chr17 is related to the micro-autolysis of cells and may be closely related to the resistance of plants to root-knot nematodes (Avci et al., 2010).

The salt tolerance traits of grapes obtained the most significant correlations among the six rootstock resistance traits. The main hazards caused by salt stress are osmotic stress and ion toxicity (Munns and Tester, 2008). An important approach for plants to deal with salt stress is to regulate the ion balance in the cell and maintain a high K^+^/Na^+^ in the cytoplasm (Munns, 2002). Na^+^ is excreted to the outside of the cell through membrane Na^+^/H^+^ exchange or antiporters on the plasma membrane. Conversely, excessive Na^+^ is transported to the vacuole through vacuolar Na^+^/H^+^ exchange or antiporters for storage to reduce the toxicity of Na^+^ ions, and as an osmotic regulator, reduce the cell osmotic potential (Apse et al., 1999). Fortunately, the sodium/hydrogen exchanger 2 gene was also identified, and multiple cysteine-rich receptor-like protein kinases may be related to the regulation of salt tolerance in grapes. To determine the activities of the candidate genes identified in this study, to be confirmed using other genotypes with opposing traits and more waterlogging stress.

## 5 Conclusion

By resequencing 77 common grape rootstock varieties, we revealed the clusters and domestication characteristics of grape rootstocks at the genomic level and analyzed the population structure of the rootstocks, which is of great significance to the improvement of rootstock varieties. The results indicated that the 77 rootstocks originated from five ancestral components, assembled into ten groups. The wild resources of *V. amurensis* and *V. davidii*, originating from China and being generally considered to have stronger resistance against biotic and abiotic stresses, were sub-divided from the other populations except *V. rotundifolia* from the United States. A large number of SNP sites were excavated, providing a possible direction for the development of SNP molecular markers for rootstocks. A high level of linkage disequilibrium was found among the 77 rootstock genotypes, GWAS analysis among the grape rootstocks located 631, 13, 9, 2, 810, and 44 SNP loci that were responsible to resistances to phylloxera, root-knot nematodes, salt, drought, cold and waterlogging traits. The findings provide a large amount of genomic data on grape rootstocks, thus offering a theoretical basis for further research on the resistance mechanism of grape rootstocks and the breeding of resistant varieties. Most importantly, these results support the huge potential of China’s wild resources as a source of stress-resistance factors in future breeding initiatives to cope with climate changes and the increasing demand for sustainable viticulture.

## Data availability statement

The datasets presented in this study can be found in online repositories. The names of the repository/repositories and accession number(s) can be found below: https://www.ncbi.nlm.nih.gov/ , PRJNA764455.

## Author contributions

LS and JF developed the project; PW and TZ designed and performed experiments, together with ZZ; PW, ZL and TZ analyzed data; FZ, XJ measured phenotype, physiological and biochemical index; PW and TZ wrote the article, LS, TP, TZ and JF revised the article. All other authors read and contributed to previous versions and approved the final version.

## Supplementary material

Supplementary Figure 1 Seventy-seven samples of grape rootstocks. According to the source of the parent, the samples were divided into 10 groups, represented by different colors, and the samples were further divided into 5 blocks according to the region and the phylogenetic tree. Population 1 (P1) are variants of *V. riparia* and their hybrids with other populations; the offspring of *V. berlandieri*× *V. riparia* and are classified as Population 2 (P2); Population 3 (P3) represents *V. rupestris* and its hybrid offspring with *V. berlandieri*; P4 represents a relatively complex group with relatively distant genetic relationships; P5 is divided into *V. rotundifolia* of American origin and the wild resources of China.

Supplementary Figure 2 Summary statistics of identity-by-descent (IBD) values calculated from all of the rootstock accessions.

Supplementary Figure 3 Population structure of 77 grape rootstocks estimated by ADMIXTURE. Each color represents one ancestral population. Each group is represented by a bar, and the length of each colored segment in the bar represents the proportion contributed by that ancestral population. The K value represents the number of the assumed ancestral population.

Supplementary Figure 4 Cross-validation (CV) error value for different model *K.*

Supplementary Figure 5 Selection elimination and gene enrichment analysis of Chinese population and other groups. *F_ST_* distribution map of eight groups (the x-axis represents different chromosomes, the y-axis represents the *F_ST_* value in the corresponding chromosome window, and the dotted line represents the selection threshold top 5%); GO term and KEGG pathway enrichment of the candidate selective sweep genes (the size of the bubble represents the number of genes in the corresponding GO category, the color of the bubble shows the corresponding *P-*value, and the rich factor shows the percentage of enriched genes out of the total number in the GO category).
