# Supplementary figures and images for: Whole-genome re-sequencing, diversity analysis, and stress-resistance analysis of 77 grape rootstock genotypes
Source: Front Plant Sci. 2023 Feb 9;14:1102695. doi: 10.3389/fpls.2023.1102695 (PMC9947647; doi:10.3389/fpls.2023.1102695)

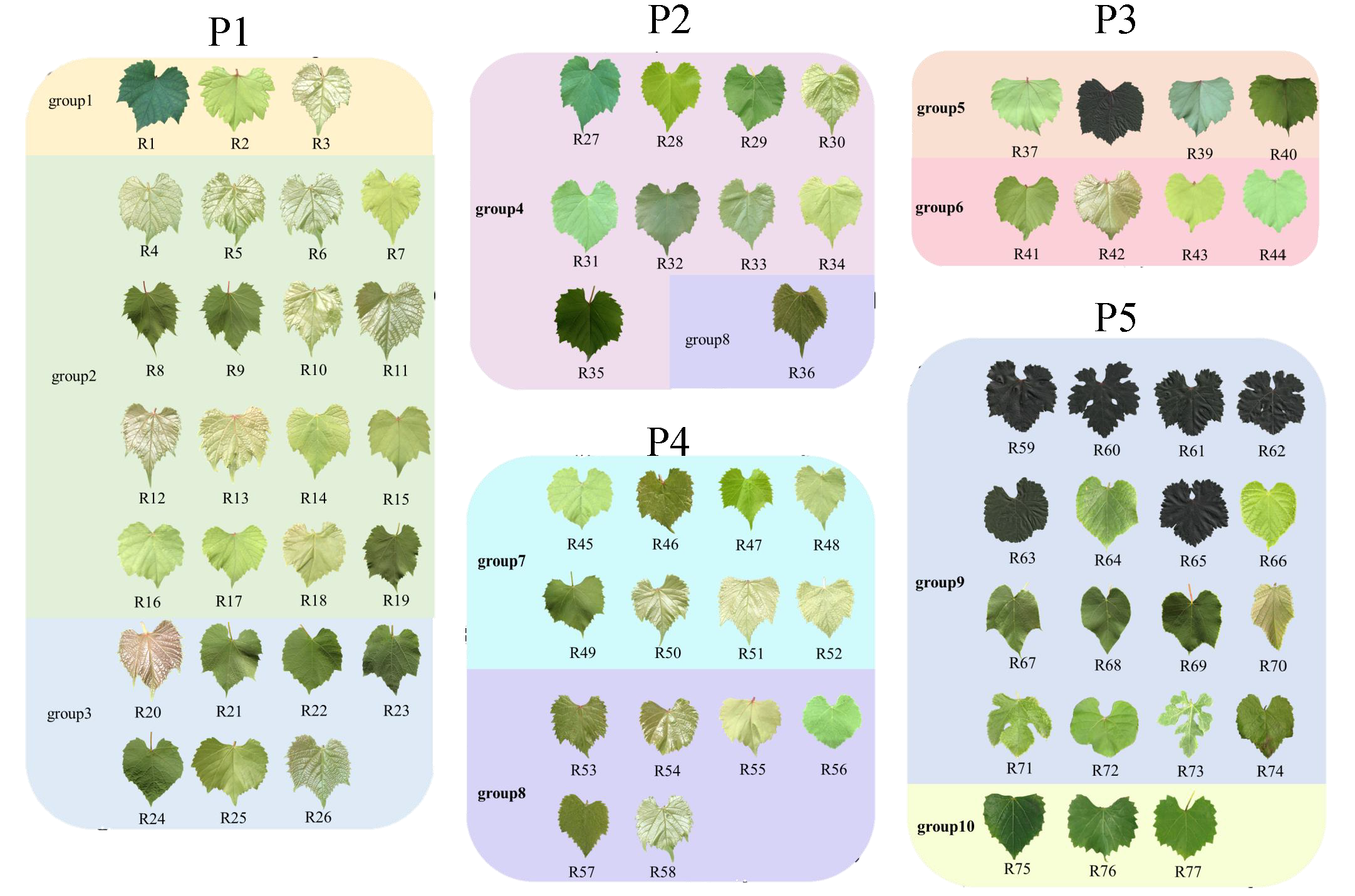

Supplement: Supplementary Figure 1 — Seventy-seven samples of grape rootstocks. According to the source of the parent, the samples were divided into 10 groups, represented by different colors, and the samples were further divided into 5 blocks according to the region and the phylogenetic tree. Population 1 (P1) are variants of V. riparia and their hybrids with other populations; the offspring of V. berlandieri× V. riparia and are classified as Population 2 (P2); Population 3 (P3) represents V. rupestris and its hybrid offspring with V. berlandieri; P4 represents a relatively complex group with relatively distant genetic relationships; P5 is divided into V. rotundifolia of American origin and the wild resources of China. [file DataSheet_1.zip › Figure S1.TIFF]

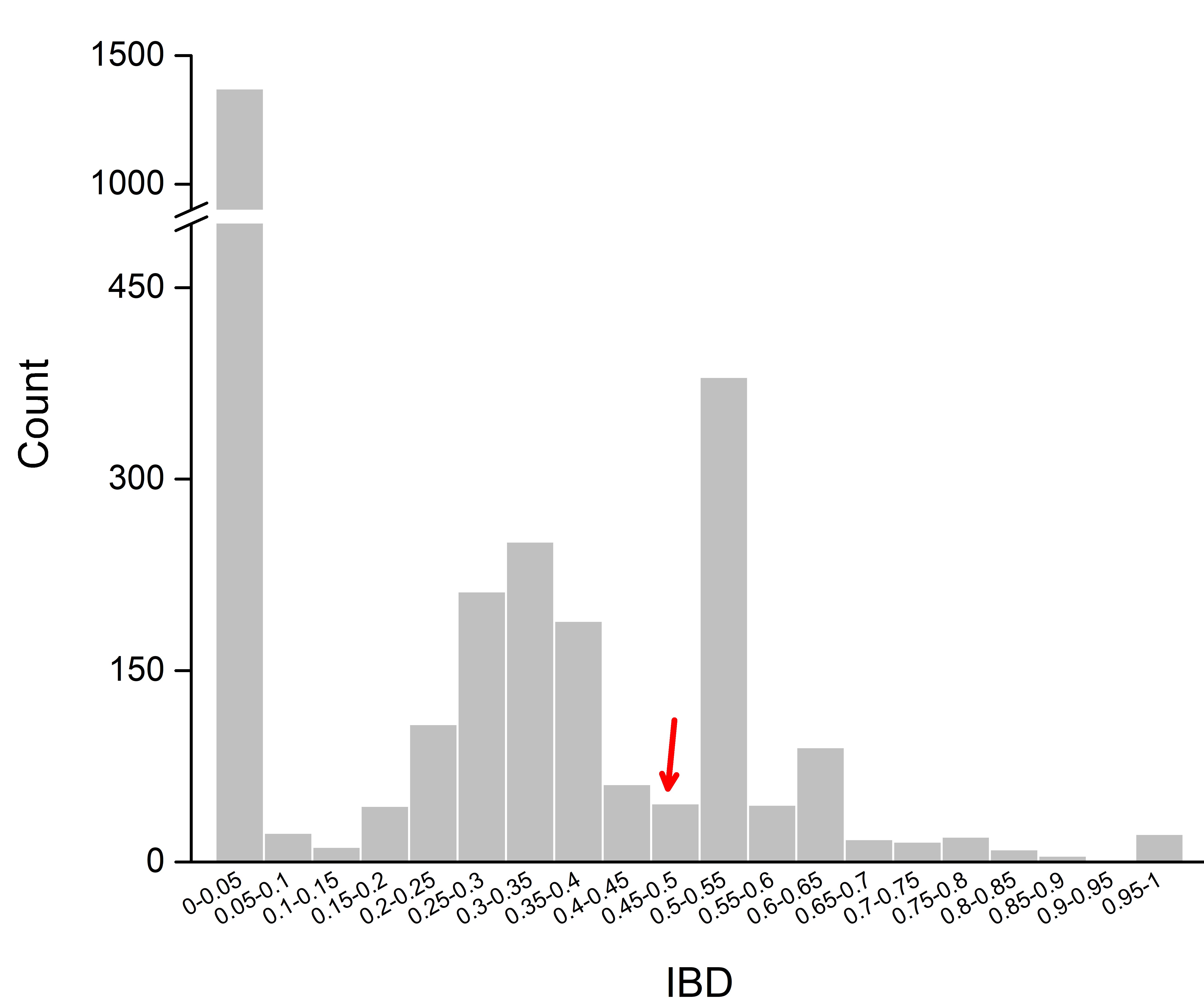

Supplement: Supplementary Figure 1 — Seventy-seven samples of grape rootstocks. According to the source of the parent, the samples were divided into 10 groups, represented by different colors, and the samples were further divided into 5 blocks according to the region and the phylogenetic tree. Population 1 (P1) are variants of V. riparia and their hybrids with other populations; the offspring of V. berlandieri× V. riparia and are classified as Population 2 (P2); Population 3 (P3) represents V. rupestris and its hybrid offspring with V. berlandieri; P4 represents a relatively complex group with relatively distant genetic relationships; P5 is divided into V. rotundifolia of American origin and the wild resources of China. [file DataSheet_1.zip › Figure S2.JPEG]

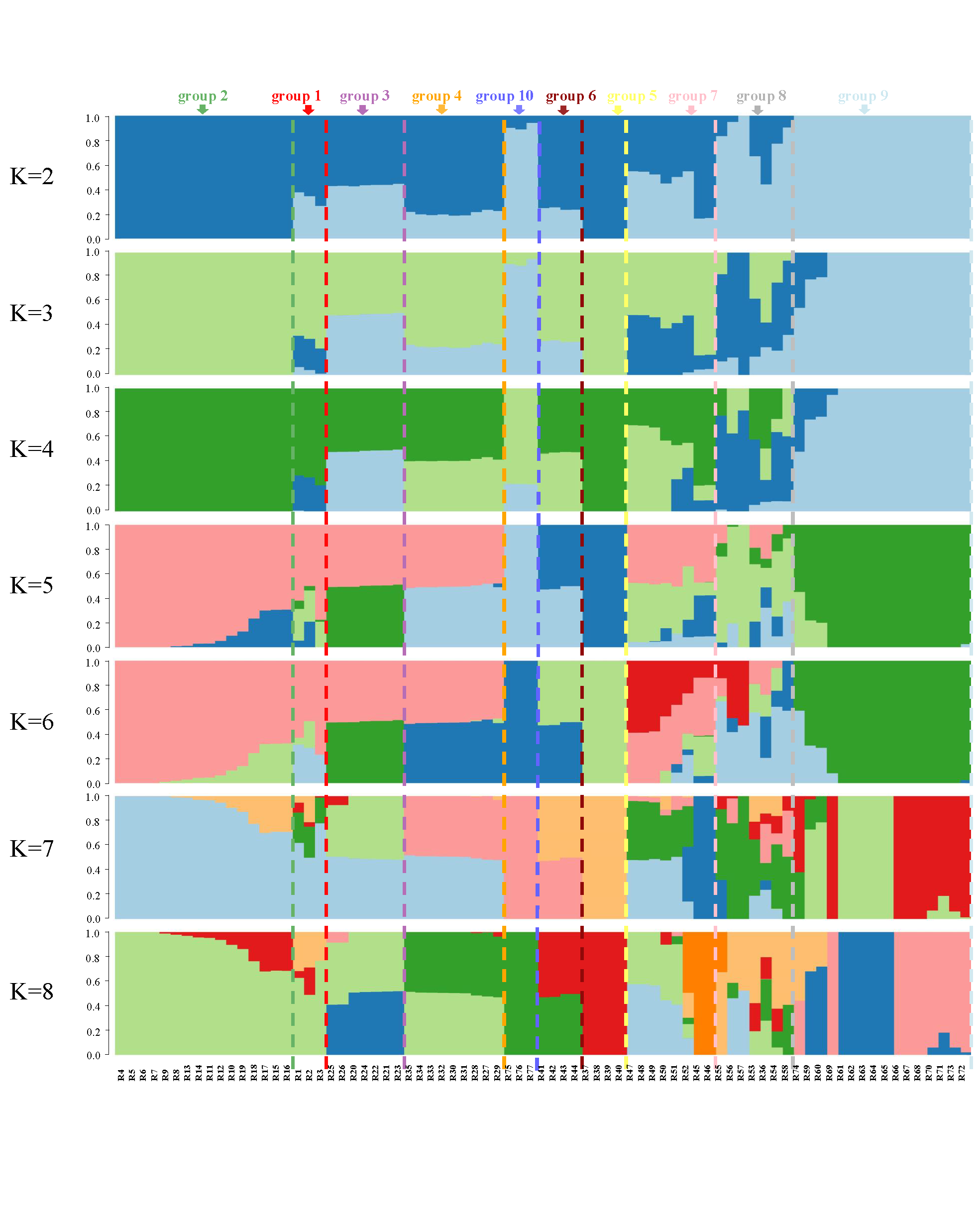

Supplement: Supplementary Figure 1 — Seventy-seven samples of grape rootstocks. According to the source of the parent, the samples were divided into 10 groups, represented by different colors, and the samples were further divided into 5 blocks according to the region and the phylogenetic tree. Population 1 (P1) are variants of V. riparia and their hybrids with other populations; the offspring of V. berlandieri× V. riparia and are classified as Population 2 (P2); Population 3 (P3) represents V. rupestris and its hybrid offspring with V. berlandieri; P4 represents a relatively complex group with relatively distant genetic relationships; P5 is divided into V. rotundifolia of American origin and the wild resources of China. [file DataSheet_1.zip › Figure S3.TIFF]

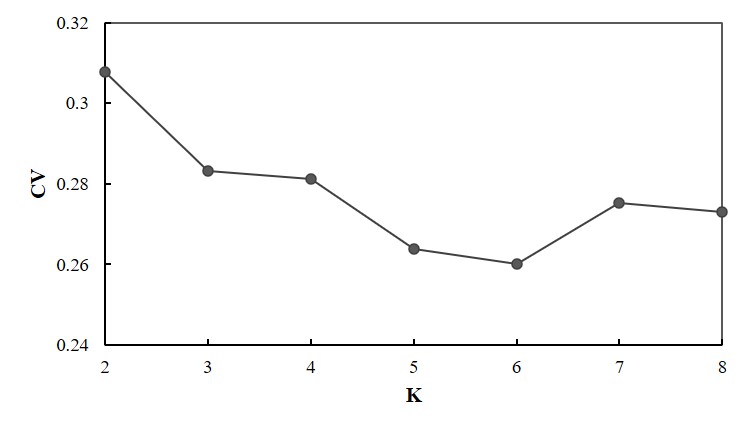

Supplement: Supplementary Figure 1 — Seventy-seven samples of grape rootstocks. According to the source of the parent, the samples were divided into 10 groups, represented by different colors, and the samples were further divided into 5 blocks according to the region and the phylogenetic tree. Population 1 (P1) are variants of V. riparia and their hybrids with other populations; the offspring of V. berlandieri× V. riparia and are classified as Population 2 (P2); Population 3 (P3) represents V. rupestris and its hybrid offspring with V. berlandieri; P4 represents a relatively complex group with relatively distant genetic relationships; P5 is divided into V. rotundifolia of American origin and the wild resources of China. [file DataSheet_1.zip › Figure S4.JPEG]

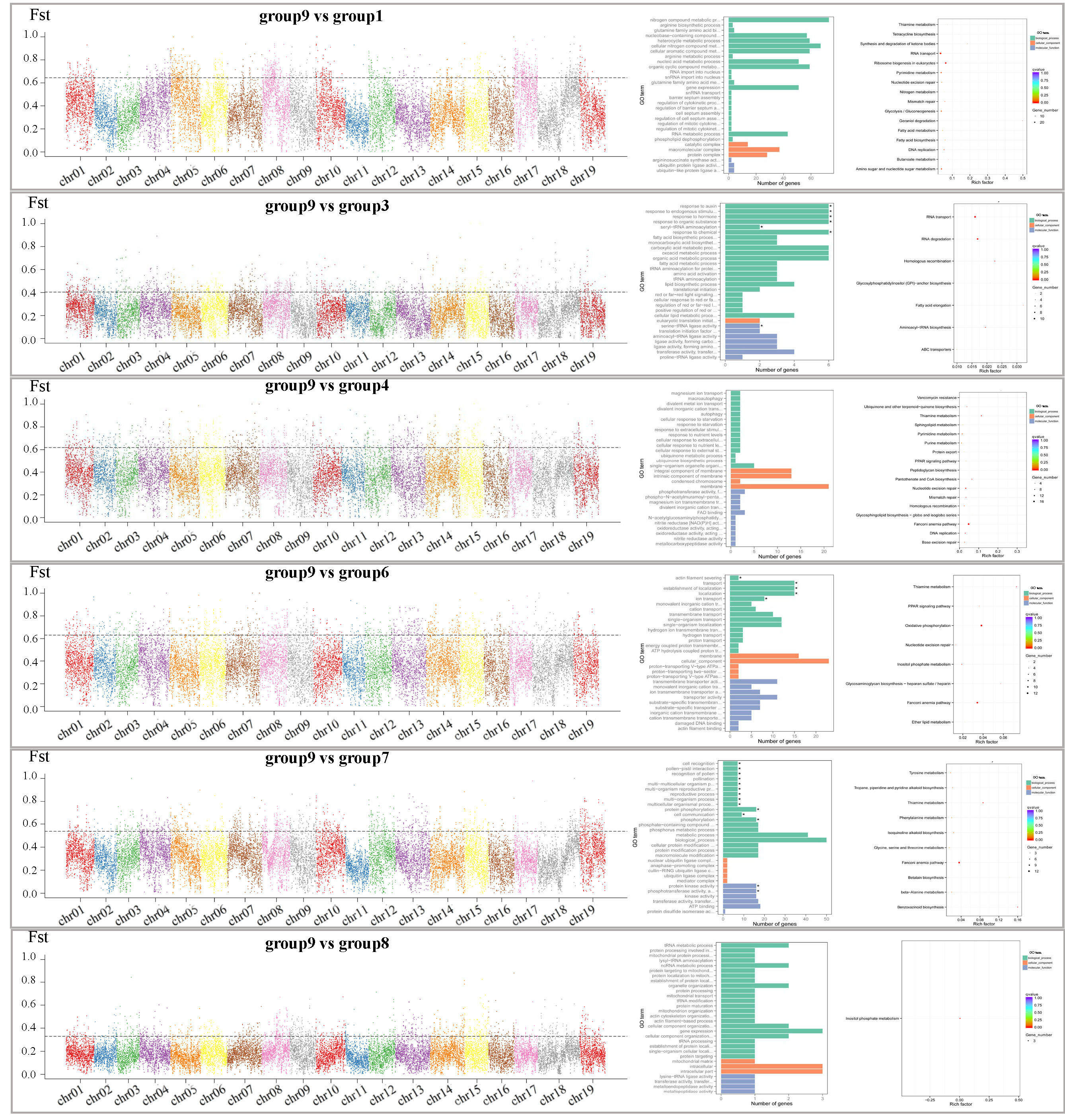

Supplement: Supplementary Figure 1 — Seventy-seven samples of grape rootstocks. According to the source of the parent, the samples were divided into 10 groups, represented by different colors, and the samples were further divided into 5 blocks according to the region and the phylogenetic tree. Population 1 (P1) are variants of V. riparia and their hybrids with other populations; the offspring of V. berlandieri× V. riparia and are classified as Population 2 (P2); Population 3 (P3) represents V. rupestris and its hybrid offspring with V. berlandieri; P4 represents a relatively complex group with relatively distant genetic relationships; P5 is divided into V. rotundifolia of American origin and the wild resources of China. [file DataSheet_1.zip › Figure S5.TIFF]
